# Dynamic Modelling of Embeddable Piezoceramic Transducers

**DOI:** 10.3390/s17122801

**Published:** 2017-12-05

**Authors:** Linsheng Huo, Xu Li, Hongnan Li, Zhijie Wang, Gangbing Song

**Affiliations:** 1 Key Laboratory of Coastal and Offshore Engineering, Dalian University of Technology, Dalian 116024, China; lshuo@dlut.edu.cn (L.H.); lx3818262@163.com (X.L.); hnli@dlut.edu.cn (H.L.); 2 School of Civil Engineering, Shenyang Jianzhu University, Shenyang 110168, China; 3 School of Civil Engineering, Southwest Jiaotong University, Chengdu 610031, China; 4 Smart Materials and Structures Laboratory, Department of Mechanical Engineering, University of Houston, Houston, TX 77204, USA

**Keywords:** Lead Zirconate Titanate (PZT) transducers, embeddable PZT transducer, dynamic model of PZT transducers, effect of waterproof layer, effect of protecting layer

## Abstract

Embedded Lead Zirconate Titanate (PZT) transducers have been widely used in research related to monitoring the health status of concrete structures. This paper presents a dynamic model of an embeddable PZT transducer with a waterproof layer and a protecting layer. The proposed model is verified by finite-element method (FEM). Based on the proposed model, the factors influencing the dynamic property of the embeddable PZT transducers, which include the material and thickness of the protecting layer, the material and thickness of the waterproof layer, and the thickness of the PZT, are analyzed. These analyses are further validated by a series of dynamic stress transfer experiments on embeddable PZT transducers. The results show that the excitation frequency can significantly affect the stress transfer of the PZT transducer in terms of both amplitude and signal phase. The natural frequency in the poling direction for the PZT transducer is affected by the material properties and the thickness of the waterproof and protecting layers. The studies in this paper will provide a scientific basis to design embeddable PZT transducers with special functions.

## 1. Introduction

Piezoceramic materials, which are characterized by their low cost, fast response, wide bandwidth, effective electromechanical coupling [1,2], dual actuation and sensing capacities [3], and ease of integration with various structures [4,5], are often used to construct transducers with multiple functions [6,7,8]. The embeddable Lead Zirconate Titanate (PZT) transducers, which are fabricated by sandwiching a waterproofed PZT patch with electric wires between two protective layers, can be used as an actuator or a sensor to generate a stress wave or to sense a stress wave. The embeddable PZT transducers have been widely used in researches that are related to structural health monitoring in the civil engineering field. Gu et al. developed a smart aggregate (SA) by embedding piezoelectric transducers into a concrete block during casting, and the proposed method was used to monitor the strength of concrete structures at early ages [9]. Song et al. developed an SA based impact monitoring system to detect the collision of over-height vehicles with bridges [10]. In addition, SAs have been used in researches to detect damages in reinforced-concrete beams [11,12,13], columns [14], shear walls [15], frame structures [16], Fiber Reinforced Polymer (FRP) repaired concrete column [17], and bridges [18]. The smart aggregates were also employed to monitor the process of soil freeze and thaw [19], the very-early-age concrete hydration [20,21], the grout compactness of post-tensioning tendon duct [22], and the debonding between the steel tube and the confined concrete core of Concrete-Filled Steel Tubes (CFSTs) [23]. In addition, the smart aggregates can work as an embedded AE sensors for the health monitoring of concrete structures [24]. Recently, the wireless smart aggregate technology for concrete structural health monitoring was reported [25]. 

The embedded or surface bonded piezoelectric transducers (PZTs) can be used to monitor the curing progression and to detect applied stress, damage onset, and damage propagation in concrete, by means of the electromechanical impedance (EMI) method [26,27,28]. The Combination of smart aggregates with surface bonded PZTs for structural health monitoring can provide an effective way to assess both the local and overall conditions of the structure [29]. The embedded transducers are especially adapted for online ultrasonic monitoring, due to their low cost, small size, and broad frequency band [30]. Yang et al. developed a reusable PZT transducer setup for the monitoring of the initial hydration of concrete and structural health, and the impedance analyzer was used to acquire the admittance signatures of the PZT [31]. Annamdas et al used the electromechanical impedance (EMI)-based technique with piezoceramic (PZT) sensors to monitor the underground support structures [32]. Yang et al. proposed the comprehensive method of health monitoring and damage assessment of rocks, employing smart optical fiber sensor (OFS) and piezoelectric impedance sensor [33]. Yang et al. proposed a sub-frequency interval approach in electromechanical impedance technique for the health monitoring of concrete structure by dividing the large frequency (30–400 kHz) range into sub-frequency intervals and calculating their respective root mean square deviation (RMSD) values [34]. 

Stress waves are often used in structural damage detection and health monitoring [35,36,37,38], and PZT transducers are commonly used for generating and detection stress waves. The mechanical model of the embedded PZT transducer is extremely important in manufacturing these transducers for the damage detection based on the response signal of PZT transducers. An electromechanical impedance (EMI) model for the health monitoring of cylindrical shell structures was presented and experimentally verified [39]. An extended formulation of an eighteen-node assumed strain three-dimensional element was proposed to analyze fully coupled mechanical-electric problems [40]. The viscous damping and shear strain correction factors were introduced in an analytical electro-mechanical model of a piezoelectric transducer [41]. An efficient electromechanically coupled geometrically nonlinear zigzag theory was developed for the buckling analysis of hybrid piezoelectric beams under electro-thermo-mechanical loads [42]. A parametric model for PZT embedded in the concrete was proposed, and the stress response that was caused by the drying shrinkage of concrete, temperatures, embedded depth, embedded materials, and thickness of the waterproof layer was analyzed [43,44,45]. The stress distribution in SAs was investigated to compute the sensitivity of the seismic compressive stress and shear stress in concrete structures [46,47]. A finite-element approach to model the nonlinear behavior of piezo-integrated structures was developed, and the constitutive relations were extended to include quadratic and cubic nonlinear terms [48]. A modeling approach was proposed to analyze the system stiffness and natural frequency behavior of a distributed compliant mechanism with embedded PZT actuators, using a general-purpose finite-element system [49]. The electromechanical coupling properties that were caused by the packaging manner and position of PZT were analyzed for cement-based PZT transducers [50]. The sensitivity of a type of embedded active PZT sensor in structural impact damage detection was studied via theoretical and experimental analyses, and a new embedded two-dimensional (2D) electromechanical impedance model, in which the PZT patch can be protected from external impacts or disturbances, was formulated [51]. The finite-element method was applied to study the piezoelectric performance of PZT and to evaluate the effective properties of piezoelectric fiber composites [52]. Madhav and Soh proposed the embedded PZT–structure interaction model to extract the mechanical impedance of the PZT patch embeddable plane structure [53]. 

In addition, a PZT transducer can be used as an energy harvesting device [54,55,56] and lead to the development of a structural damage identification technique that is based on the impedance method [57,58,59]. Many different electromechanical impedance models have been proposed to characterize the interaction between a PZT transducer and the host structure [60,61,62,63,64]. Karayannis et al. used the signals of the piezoelectric transducer to predict the forthcoming failures at early damage stages and the proposed an integration analytical approach based on the electromechanical admittance [65]. A portable real-time Wireless impedance/Admittance Monitoring System (WiAMS) in damage diagnosis of shear-critical RC beams was experimentally studied [66,67]. The effects of temperature and load on impedance and conductance spectra of the sensor were investigated with impedance-based approach by Xu et al. [68], and the root mean square deviation (RMSD) index was employed to intuitively indicate the impedance variation of the embedded PZT sensor under temperature and load. The model of the electromechanical impedance (EMI) of piezoceramics transducers was further developed and applied to aircraft structural health monitoring [69].

The effect of the bond layer on the electro-mechanical response of a smart system was experimentally studied, and the effects of shear lag due to the finite thickness bond layer were successfully identified [70]. Ong et al. proposed a one-dimensional electro-mechanical (EM) impedance model to account for the shear lag between the PZT patch and the host structure [71]. Madhav and Soh presented a three-dimensional (3D) interaction model of a PZT-structure, which considers the mass of both the PZT transducers and the adhesive, and experimentally verified its correctness [72]. 

Embedded PZT transducers not only detect the internal stress of civil structures, but also have the capacity to detect internal damages by emitting and detecting stress waves, whose frequencies are considerably higher than that of the structural vibration. Therefore, it is essential to study the dynamic property of embedded PZT transducers and assure the relationship between the stress and the output signal of transducers in different frequency ranges [73]. However, current mechanical models of embedded PZT transducers are limited in that they neglect the effect of the excitation frequency on the mechanical model. 

When considering the limitation of the aforementioned mechanical models, this paper presents a dynamic stress transfer model for embedded PZT transducers that is achieved by considering a PZT transducer with a structure of a waterproof layer and a protecting layer. The proposed model is validated using the finite-element method. The effects of the excitation frequencies, material property and thickness of the protecting layer and waterproof layer, and thickness of the PZT transducer on the dynamic performance of the embedded PZT transducers are analyzed. Finally, the proposed model is validated via a series of experiments. 

## 2. Dynamic Model of the Embeddable PZT Transducer

**Figure 1:**
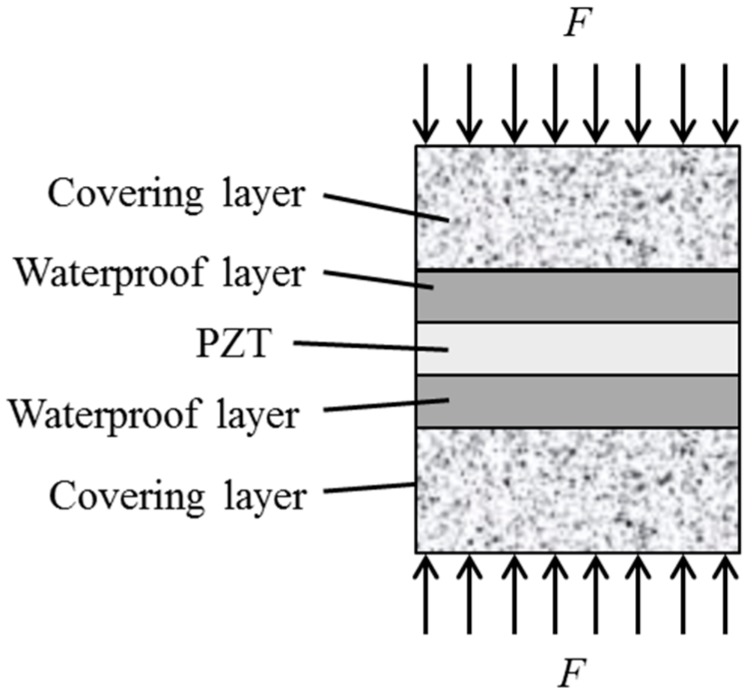
Schematic of the embeddable Lead Zirconate Titanate (PZT) transducer.

An embeddable PZT transducer used in the damage detection of concrete structures can be characterized as a PZT patch with water proof layers and protection (covering) layers, as shown in Figure 1. Such an embeddable PZT transducer can be typically applied to monitor the stress or detect the elastic wave that propagates in the structures. 

The function of the waterproof layer is to offer electric protection of the PZT patch, whereas the protecting (covering) layer offers mechanical protection to the fragile PZT patch. The embeddable PZT transducer that is considered here tends to use the *d*_33_ mode to measure the stress and acquire the stress wave in the poling direction of *d*_33_. Therefore, two assumptions are made to derive the mechanical model of the embeddable PZT sensor:

1. The PZT patch is only subjected to the axial stress from the poling direction, and the stresses from other directions are neglected.
2. The distribution of axial stress along the poling surface is homogeneous.

**Figure 2:**
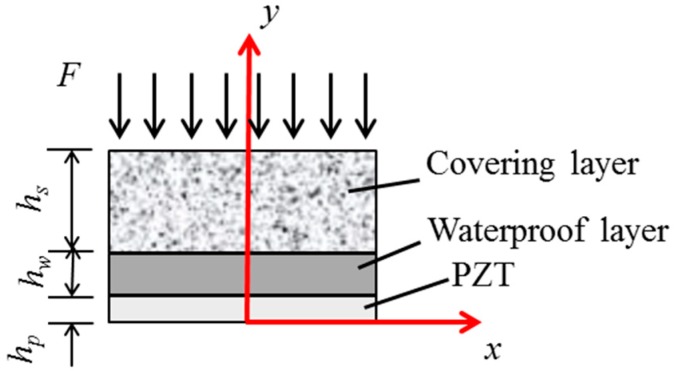
A half model of the embeddable PZT transducer.

The symmetrical component of the PZT model in Figure 2 is selected for analysis, where *h_s_* and *h_w_* are the thicknesses of the protecting layer and waterproof layer, respectively, and *h_p_* is the half thickness of the PZT patch. The wave propagation equations of the protecting layer, waterproof layer, and PZT patch are as follows:

(1) $$\begin{array}{l} E_{p}\frac{\partial^{2}u_{p}}{\partial y^{2}}=\rho_{p}\frac{\partial^{2}u_{p}}{\partial t^{2}}\\ E_{w}\frac{\partial^{2}u_{w}}{\partial y^{2}}=\rho_{w}\frac{\partial^{2}u_{w}}{\partial t^{2}}\\ E_{c}\frac{\partial^{2}u_{c}}{\partial y^{2}}=\rho_{c}\frac{\partial^{2}u_{c}}{\partial t^{2}} \end{array}$$

where *u_p_*, *u_w_* and *u_c_* are the displacement of the PZT, the waterproof layer and protecting layer along the *y* direction, respectively; *E_p_*, *E_w_,* and *E_c_* are the elastic moduli of the PZT patch, waterproof layer, and protecting layer, respectively; and, *ρ_p_*, *ρ_w,_* and *ρ_c_* are the densities of the PZT patch, waterproof layer, and protecting layer, respectively.

When the external force is a harmonic vibration *F*_0_*e^iωt^*, only the steady state is considered. Then, the displacements of the PZT, waterproof layer and protecting layer along the *y* direction are *U_p_ e^iωt^*, *U_w_ e^iωt^* and *U_c_ e^iωt^*, respectively. Equation (1) becomes,

(2) $$\begin{array}{l} E_{p}\frac{d^{2}U_{p}}{\partial y^{2}}+\rho_{p}\omega^{2}U_{p}=0\\ E_{p}\frac{d^{2}U_{p}}{dy^{2}}+\rho_{p}\omega^{2}U_{p}=0\\ E_{c}\frac{d^{2}U_{c}}{dy^{2}}+\rho_{c}\omega^{2}U_{c}=0 \end{array}$$

The general solutions of *U_p_*, *U_w_* and *U_c_* are,

(3) $$\begin{array}{c} U_{P}=C_{1}\sin(\Gamma_{p}y)+C_{2}\cos(\Gamma_{p}y)\\ U_{w}=C_{3}\sin(\Gamma_{p}y)+C_{4}\cos(\Gamma_{p}y)\\ U_{c}=C_{5}\sin(\Gamma_{p}y)+C_{6}\cos(\Gamma_{p}y) \end{array}\}$$

where *Γ_p_* = *ρ_p_ω*^2^/*E_p_*, *Γ_w_* = *ρ_w_ω*^2^/*E_w_* and *Γ_c_* = *ρ_c_ω*^2^/*E_c_*. The boundary conditions for the PZT, the waterproof layer, and the protecting layer are,

(4) $$U_{p}=0,\;for\;y=0$$

(5) $$\begin{array}{c} U_{p}=U_{w}\\ E_{p}\frac{dU_{p}}{dy}=E_{w}\frac{dU_{w}}{dy} \end{array}\}\;for\;y=h_{p}$$

(6) $$\begin{array}{c} U_{w}=U_{c}\\ E_{w}\frac{dU_{w}}{dy}=E_{c}\frac{dU_{c}}{dy} \end{array}\}\;for\;y=h_{p}+h_{w}$$

(7) $$E_{c}A\frac{dU_{c}}{dy}=F_{0}\;for\;y=h_{p}+h_{w}+h_{s}$$

where *A* is the area of the PZT poling surface and *ε*_0_ is defined as *F*_0_/(*E_s_A*). The following linear system of equations is obtained by substituting the above boundary conditions into Equation (3),

(8) $$\left[\begin{array}{cccccc} \sin0 & \cos0 & 0 & 0 & 0 & 0\\ \sin(\Gamma_{p}h_{1}) & 0 & -\sin(\Gamma_{w}h_{1}) & -\cos(\Gamma_{w}h_{1}) & 0 & 0\\ E_{p}\Gamma_{p}\cos(\Gamma_{p}h_{1}) & 0 & -E_{w}\Gamma_{w}\cos(\Gamma_{w}h_{1}) & E_{w}\Gamma_{w}\cos(\Gamma_{w}h_{1}) & 0 & 0\\ 0 & 0 & \sin(\Gamma_{w}h_{2}) & \cos(\Gamma_{w}h_{2}) & -\sin(\Gamma_{c}h_{2}) & -\cos(\Gamma_{c}h_{2})\\ 0 & 0 & E_{w}\Gamma_{w}\cos(\Gamma_{w}h_{2}) & -E_{w}\Gamma_{w}\sin(\Gamma_{w}h_{2}) & -E_{c}\Gamma_{c}\cos(\Gamma_{c}h_{2}) & E_{c}\Gamma_{c}\sin(\Gamma_{c}h_{2})\\ 0 & 0 & 0 & 0 & \Gamma_{c}\cos(\Gamma_{c}h_{3}) & -\Gamma_{c}\sin(\Gamma_{c}h_{3}) \end{array}\right]\left[\begin{array}{c} C_{1}\\ C_{2}\\ C_{3}\\ C_{4}\\ C_{5}\\ C_{6} \end{array}\right]=\left[\begin{array}{c} 0\\ 0\\ 0\\ 0\\ 0\\ 0 \end{array}\right]$$

The values of constants *C*_1_–*C*_6_ can be computed by solving Equation (8). Please note that *C*_1_–*C*_6_ all have the parameter *ε*_0_. Then, the mode shape of the PZT transducer is expressed as,

(9) $$\frac{dU_{p}}{dy}=C_{1}\Gamma_{p}\cos(\Gamma_{p}y)$$

The strain along the *y* direction *ε_p_* is derived as,

(10) $$\varepsilon_{p}=\frac{2}{h_{p}}\int_{0}^{h_{p}}\frac{dU_{p}}{dy}dye^{i\omega t}=\frac{2}{h_{p}}C_{1}\sin(\Gamma_{p}h_{p})e^{i\omega t}$$

There is a linear relationship between the output voltage and the strain along the poling direction of the PZT patch. The output voltage of PZT can be expressed as,

(11) $$V=\frac{\varepsilon_{p}d_{33}A}{s_{33}C}$$

where *d*_33_ is the piezoelectric constant of the PZT transducer; *S*_33_ is the elastic coefficient of the PZT transducer; and, *C* is the capacitance of the PZT transducer. 

The strain transfer mechanism along the poling direction of the PZT transducer must be considered to analyze the dynamic property of the transducer.

## 3. Numerical Simulation

To analyze the dynamic stress transfer property of an embeddable piezoceramic transducer, the finite element method (FEM) is used to compute the stress transfer process for the PZT-waterproof-layer-protecting-layer structure that is subjected to a harmonic excitation at different frequencies by ANSYS software. The plane82 element is used to simulate the PZT patch, waterproof layer, and the protecting layer. All parameters of three layers are shown in Table 1, and the width of PZT transducer is 0.01 m. The half model of the symmetrical structure in Figure 2 along the *y* axis is analyzed. For boundary conditions of the model, the displacement in the *x* direction is restrained when *x* is equal to 0, and the displacement in the *y* direction is constrained when *y* is equal to 0. 

**Figure 3:**
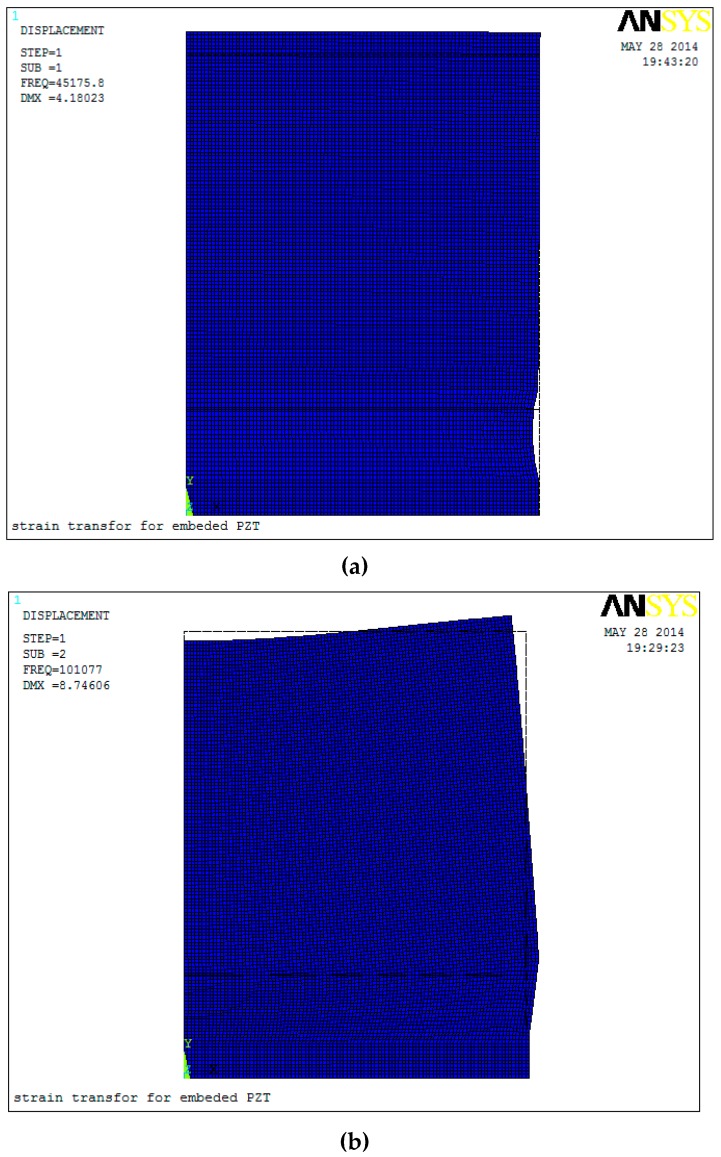

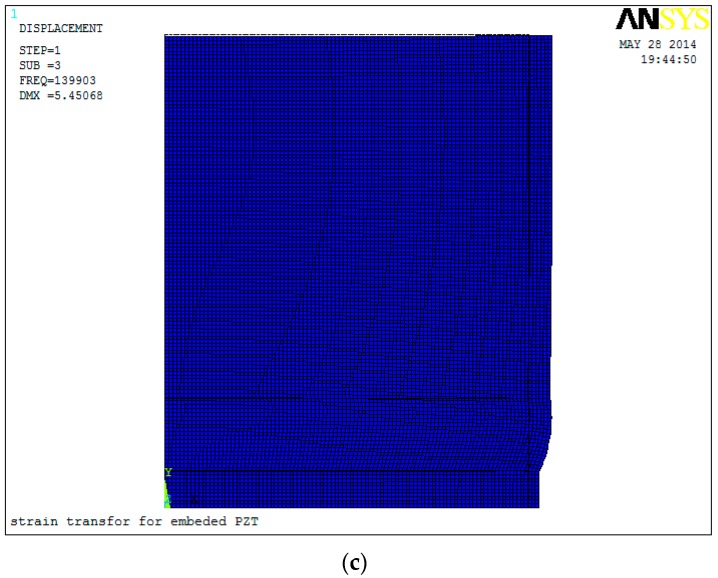
First three mode shapes of the embeddable PZT sensor. (**a**) The 1st modal shape; (**b**) The 2nd modal shape; and, (**c**) The 3rd modal shape.

First, the modal analysis is performed. The first three modal frequencies are 48.18, 101.08, and 139.9 kHz. The first three mode shapes are shown in Figure 3. The first modal shape is the vibration along the *y* direction, the second modal shape is the twisting vibration along the *z* axis, and the third modal shape is the stretching vibration along the *x* direction. Since the PZT patch mainly depends on the piezoelectric constant *d*_33_, the shearing and twisting components along the poling direction only affect the output voltage slightly. Therefore, the previous assumptions are reasonable in this paper. 

**Figure 4:**
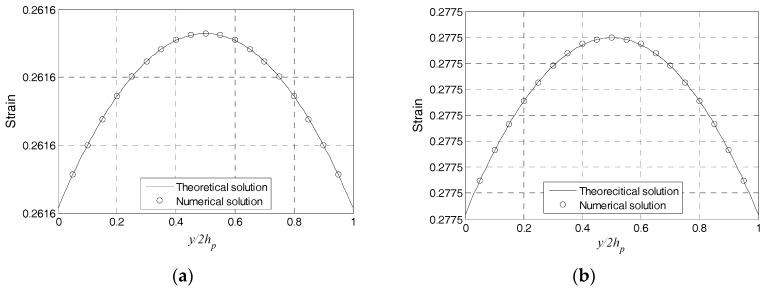

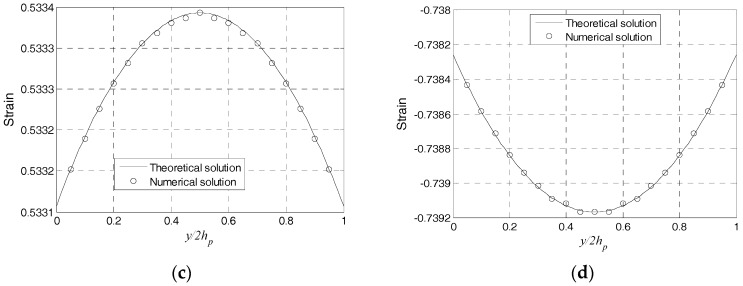
Theoretical and numerical solutions of the PZT strain distribution along the *y* direction. (**a**) The excitation frequency is 1 kHz; (**b**) The excitation frequency is 10 kHz; (**c**) The excitation frequency is 30 kHz; and, (**d**) The excitation frequency is 50 kHz.

Then, the harmonic response analysis is performed to simulate the PZT transducer when it is subjected to the harmonic exciting. The force with the amplitude of 0.02 N is loaded in y direction, and the frequency range is from 0 to 50 kHz. Figure 4 presents the comparison between the numerical simulation results and theoretical ones for excitation frequencies of 1, 10, 30, and 50 kHz. The numerical and theoretical results are consistent, which demonstrates the rationality of the proposed method. 

In addition, according to above analysis, it can be seen that the PZT strain distributed along *y* direction gradually increases when the excitation frequency is increased from 1 kHz to 30 kHz, as shown in Figure 4a–c. When frequency is 50 kHz, which is beyond the first model frequency, the magnitude of strain is much larger, but the direction of strain is opposite of that in Figure 4d. The excitation frequency tends to influence the strain transfer property of PZT transducers.

## 4. Parametric Analysis

A numerical example is used to illustrate the effect of the excitation frequency, protecting layer, waterproof layer, and PZT patch on the dynamic properties of the transducer. Table 1 shows the parameters of the embeddable PZT transducer. The protecting and waterproof layers are made of marble and epoxy resin, respectively. The harmonic excitation *e^iωt^* is loaded along the *y* direction. The change in strain amplitude is analyzed. 

### 4.1. Effect of the Excitation Frequency

**Figure 5:**
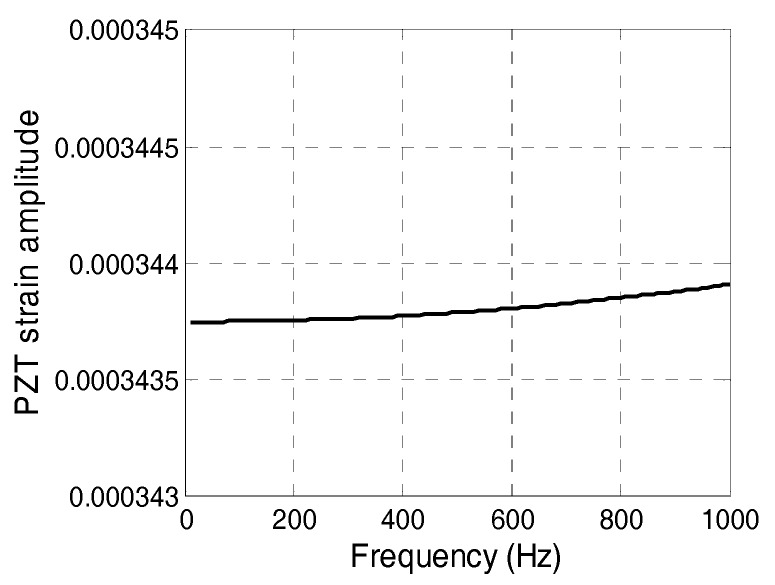
Frequency-PZT strain amplitude curve (0 Hz–1 kHz).

Figure 5 shows the change in strain amplitude when the driving frequency increased from 0 to 1 kHz. The strain amplitude is 3.438 × 10^−4^ when the driving frequency is zero. The strain amplitude increases to 3.439 × 10^−4^ when the driving frequency increased by 1 kHz. The strain amplitude only increases by 0.03% when the driving frequency increased from 0 to 1 kHz, which is within the scope of the natural frequency of civil structures. Therefore, the driving frequency does not considerably affect the amplitude of the PZT sensor, and the relationship between the output voltage and strain can be expressed by a constant of sensitivity.

**Figure 6:**
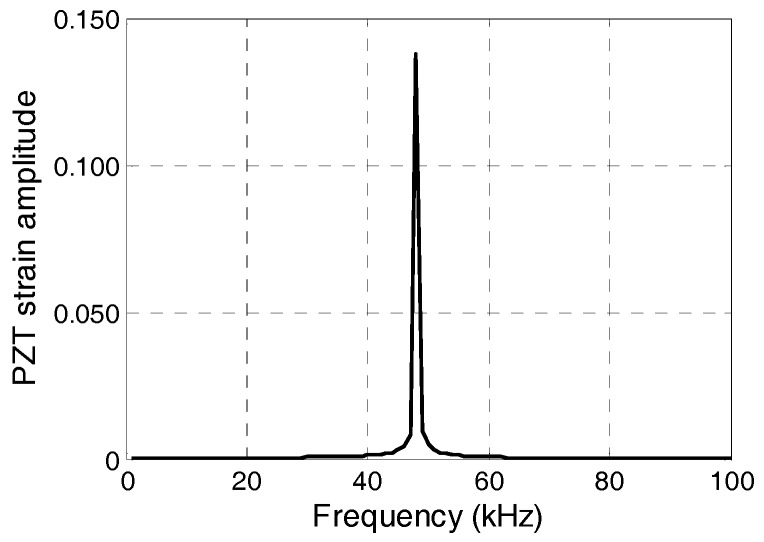
Frequency-PZT strain amplitude curve (1 kHz–100 kHz).

Figure 6 shows the change in strain amplitude when the driving frequency increased from 1 kHz to 100 kHz. The first natural frequency of the PZT sensor system is 48 kHz. The strain amplitude of the PZT patch increases dramatically when the loading frequency approaches 48 kHz. The relationship between the signal amplitude and strain cannot be expressed by a constant value of sensitivity. In addition, the PZT sensor is more likely to receive a stress wave signal of higher frequency than a vibration signal of lower frequency. 

**Figure 7:**
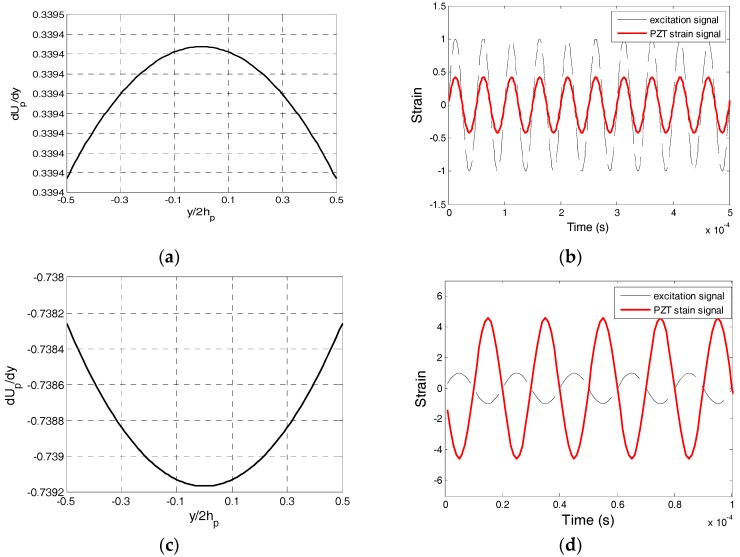
*dU_p_*/*dy* distribution along the *y* direction, the excitation signal and the PZT strain signal. (**a**) Distribution of *dU_p_/dy* when the excitation frequency is 20 kHz; (**b**) PZT strain signal and excitation signal when the excitation frequency is 20 kHz; (**c**) Distribution of *dU_p_/dy* when the excitation frequency is 50 kHz; and, (**d**) PZT strain signal and excitation signal when the excitation frequency is 50 kHz.

The driving frequency also affects the phase of the output signal. Figure 7 shows the distribution of *dU_p_*/*dy* along the *y* direction and the loading signal and output PZT signal. When the driving frequency is less than the natural frequency of the PZT transducer along the poling direction, then *dUp*/*dy* is larger than 0, as shown in Figure 7a, and the vibration directions of the PZT transducer and loading are identical, as shown in Figure 7b. However, when the driving frequency is larger than the natural frequency of the PZT sensor along the poling direction, *dUp*/*dy* is less than 0, as shown in Figure 7c, and the vibration directions of PZT and loading are opposite, as shown in Figure 7d. When the signal phase is measured, the phase difference that is caused by the vibration mode is not negligible. In addition, it is assumed that there are two PZT sensors at the same locations with natural frequencies *ω_A_* and *ω_B_*; when the excitation frequency is between *ω_A_* and *ω_B_*, there will be a phase difference of *π* between two PZT sensors.

### 4.2. Effect of the Protecting Layer

**Figure 8:**
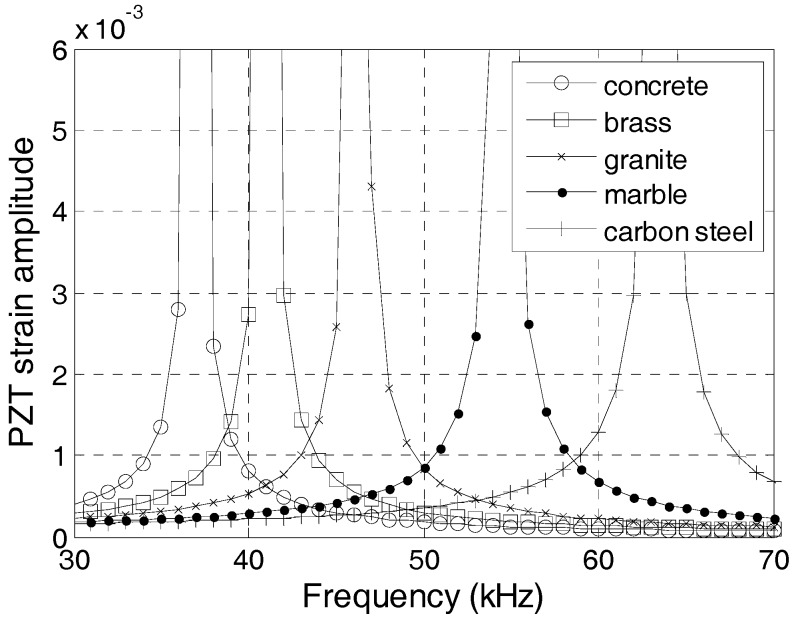
Frequency-strain curve with different protecting layer materials.

The protecting layers of the embeddable PZT transducers can be made from many types of materials. In this section, the effect of the protecting layer on the dynamic property is analyzed. The material properties of different protecting layers are listed in Table 2, where *E_c_*/*ρ_c_* = *c*^2^ and *c* is the longitudinal wave velocity of the material. Figure 8 shows the change in strain amplitude with different frequencies. The first natural frequencies for PZT sensors increase with the increases in the longitudinal wave velocity of the protecting layer materials. Therefore, the PZT sensor with a carbon steel protecting layer has the highest natural frequency for the poling direction vibration, whereas that with a concrete protecting layer has the lowest natural frequency for the poling direction vibration.

**Figure 9:**
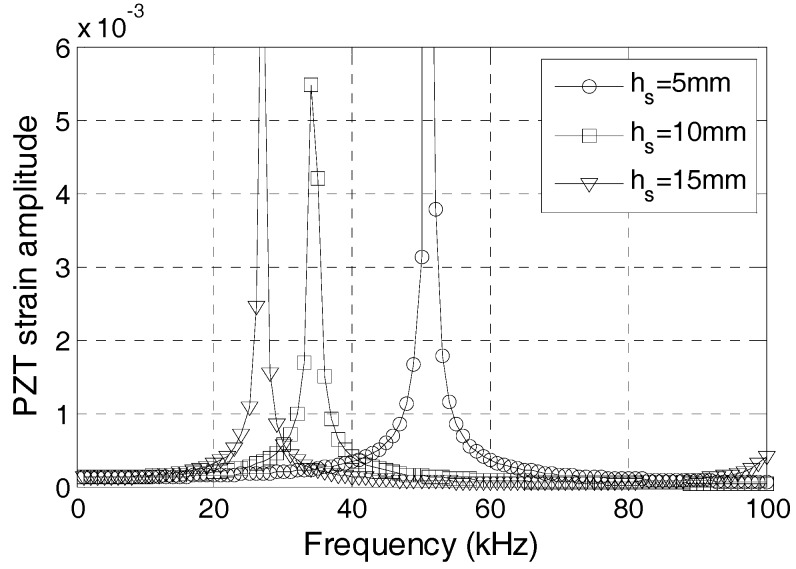
Frequency-strain curve with different protecting layer materials.

Embeddable PZT transducers are commonly made with protecting layers of different thicknesses, which can affect the dynamic property of the transducer significantly. In this section, the effect of the protecting layer thickness on the dynamic property of the transducer is studied. Figure 9 shows the PZT strain amplitude with different frequencies when the protecting layer is 5 mm, 10 mm, and 15 mm thick. A thicker protecting layer corresponds to a larger natural frequency of the sensor. Therefore, a thicker protecting layer should be used in a transducer in order to increase the natural frequency of the sensor.

### 4.3. Effect of the Waterproof Layer

**Figure 10:**
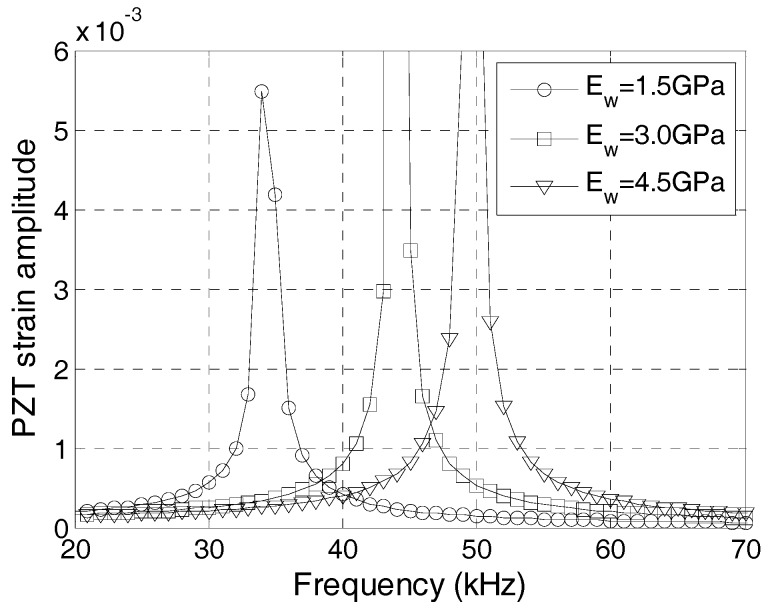
Frequency-strain curve with different water-proof layer materials.

In general, epoxy resin materials, which have a shear modulus of 1–5 GPa, are used as the waterproof layer of SAs. Because the density range of epoxy resin materials is small, an epoxy resin material with a larger elastic modulus has a higher longitudinal wave velocity. Figure 10 is the frequency-strain amplitude curve with different waterproof layer materials, where the density of the waterproof layer is 1105 kg/m^3^ and the elastic modulus is 1.5, 3, and 4.5 GPa. The natural frequency of the PZT sensor increases with the increases of the elastic modulus of the waterproof layer.

**Figure 11:**
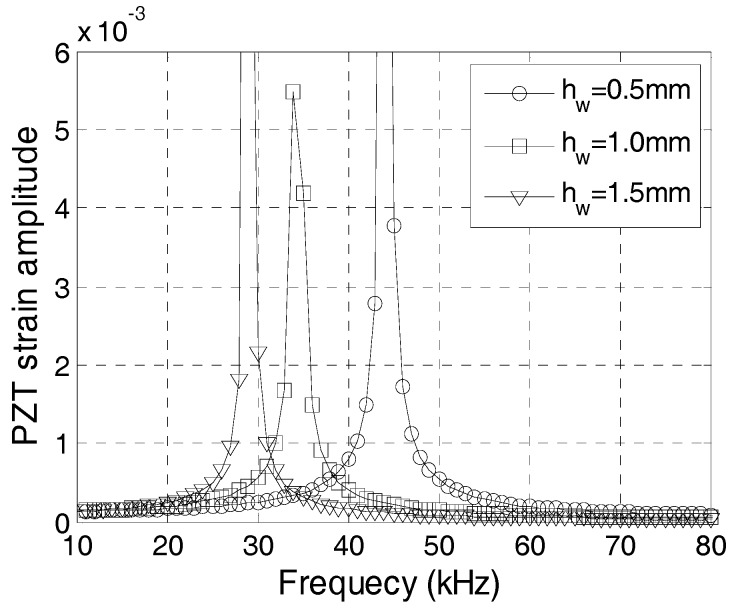
Frequency-strain curve with different waterproof layer thicknesses.

For the safety and durability of a PZT sensor, the water proof/protecting layer is necessary. Although the magnitude of signal greatly reduces if layers of water proof/protecting layers are used, PZT sensors can be manufactured with waterproof layers of different thicknesses, according to the results of this study. Figure 11 shows the PZT strain amplitude with different frequencies when the waterproof layer is 0.5 mm, 1 mm, and 1.5 mm thick. The natural frequency of the transducer increases with the decreases of the thickness of the waterproof layer. Therefore, a thinner waterproof layer should be used to achieve a higher natural frequency in the embeddable PZT transducer.

### 4.4. Effect of the PZT Thickness

**Figure 12:**
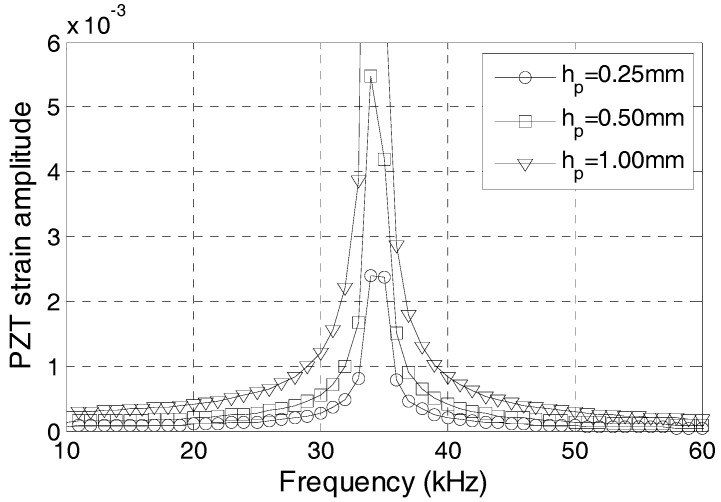
Frequency-strain amplitude curve with different waterproof layer thicknesses.

The effect of the PZT thickness on the dynamic property of the PZT transducer is studied in this section. Figure 12 shows the frequency-strain amplitude curve when the half thickness of the PZT patch is 0.25, 0.5, and 1 mm. The results confirm the minor effect of the thickness of the PZT patch on the natural frequency of the PZT sensor.

## 5. Experiment

### 5.1. Introduction

**Figure 13:**
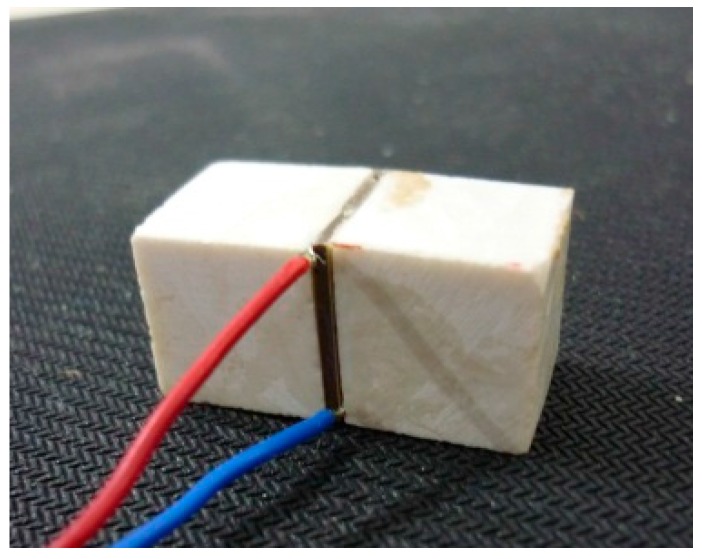
Fabricated transducer.

In this section, the stress transfer experiment that was performed on the transducer is described. The embeddable PZT transducer used in this study is shown in Figure 13. Without additional illustration, the dimensions of the protecting layer are 15 mm × 15 mm × 15 mm, and the dimensions of the PZT patch are 15 mm × 15 mm × 1 mm. The transducer was manufactured by connecting wires on the poling surface of the PZT patch, covered with epoxy resin as the waterproof layer and protected with the protecting layer. 

**Figure 14:**
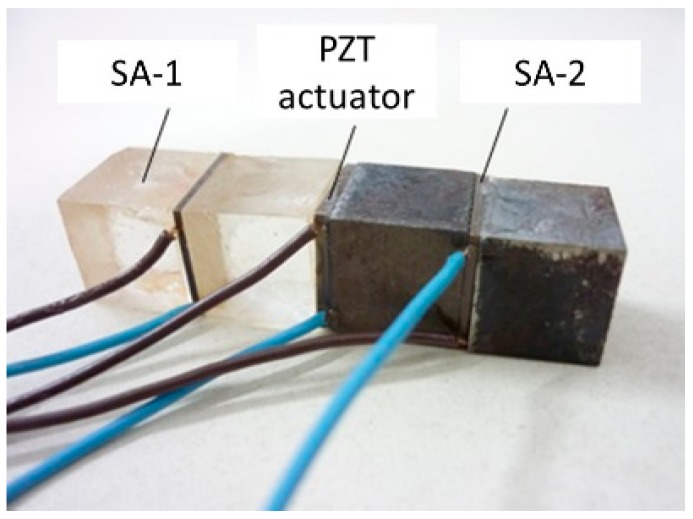
Test member.

To compare the dynamic property between two different PZT sensors, the specimen was made by bonding two transudes on both sides of the PZT patch of the actuator, as shown in Figure 14. The harmonic voltage was loaded in the actuator, and the outputs of two transducers were analyzed. The excitation device was the arbitrary waveform generator, and the oscilloscope was used as the date acquisition system, with a sampling rate of 3.13 M/s. 

**Figure 15:**
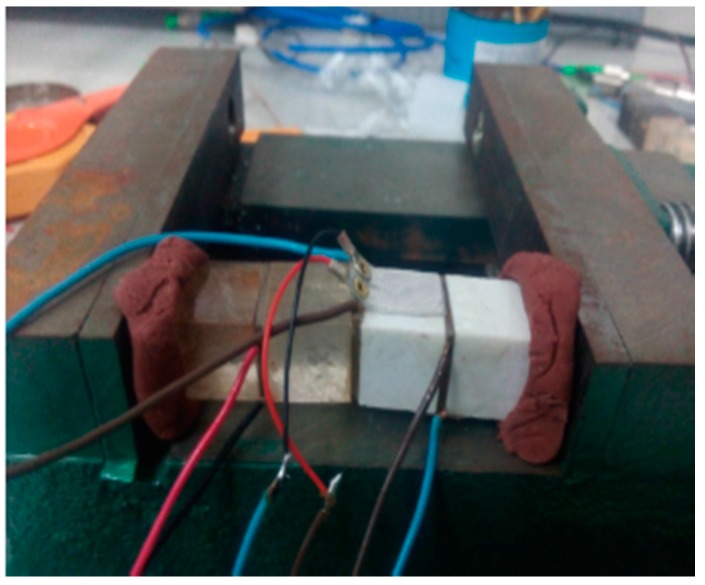
Clamping manner of specimens.

The specimen was located on the bench clamp, as shown in Figure 15, and the plasticine was used as a buffer layer between the specimen and the bench clamp such that the experiment was not affected by the clamping force from the bench clamp. The details of the specimens are provided in Table 3.

After obtaining the signal from the PZT transducers, the signal phase was extracted. The natural frequency of the transducer cannot be obtained by only the amplitude of the signals because the amplitude of the actuation force is not determined in different excitation frequencies. In this section, the natural frequency of transducer is determined by the change of phases. 

### 5.2. Effect of the Protecting Materials

**Figure 16:**
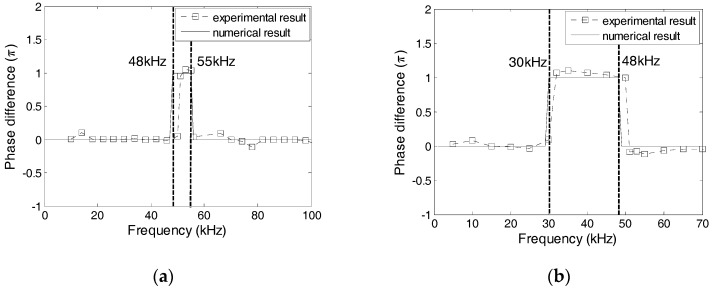
Phase difference between PZT-1 and PZT-2 from Specimens 1 and 2. (**a**) Specimen 1; (**b**) Specimen 2.

**Figure 17:**
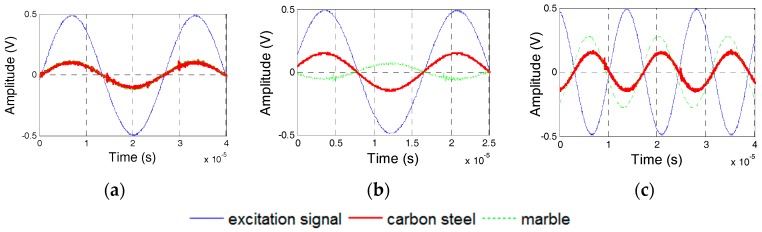
PZT transducer signal of Specimen 1. (**a**) 38 kHz Excitation Frequency; (**b**) 52 kHz Excitation Frequency; and, (**c**) 70 kHz Excitation Frequency.

**Figure 18:**
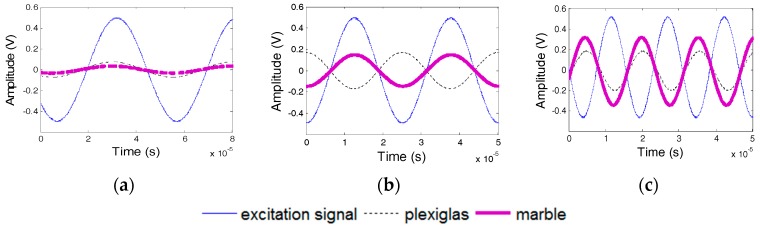
PZT transducer signal of Specimen 2. (**a**) 20 kHz Excitation Frequency; (**b**) 40 kHz Excitation Frequency; and, (**c**) 60 kHz Excitation Frequency.

The effect of the protecting layer on the dynamic property was studied using Specimens 1 and 2, where the protecting layer was manufactured from Plexiglas, marble, and carbon steel. Figure 16 shows the numerical and experimental values of the phase difference between SA-1 and SA-2 of specimens 1 and 2. The errors between the numerical and experimental values are less than 5%. In addition, the changes of phases are related to the natural frequencies of transducers. Based on Figure 16, it can be concluded that the natural frequencies of two transducers in Specimen 1 should, respectively, be 48 kHz and 55 kHz, and the natural frequencies of two transducers in Specimen 2 should, respectively, be 30 kHz and 48 kHz. Figure 17 shows the excitation signal and PZT transducer signals from transducers in Specimens 1. When the excitation frequency is 52 kHz, then the phase of the transducers with a marble protecting layer increases by *π* compared to that of the excitation signal, which indicates that the natural frequency of the transducer with a marble protecting layer is less than 52 kHz. Therefore, the natural frequency of the transducer with a marble protecting layer should be 48 kHz, and that with a carbon steel protecting layer should be 55 kHz. Similarly, Figure 18 shows that when the excitation frequency is 40 kHz, the signal phase of the transducer with the Plexiglas protecting layer increases by *π*, which indicates that the natural frequency of the transducer with a Plexiglas protecting layer should be 30 kHz, and that with a marble protecting layer should be 48 kHz. The numerical and experimental results are similar, which validates the above analysis. 

### 5.3. Effect of the Waterproof Layer Materials 

**Figure 19:**
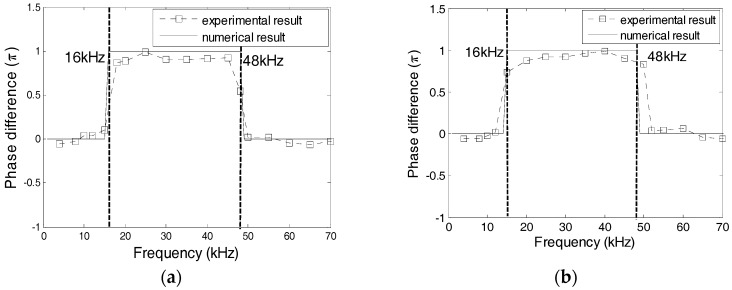
Phase difference between PZT transducers from Specimens 3 and 4. (**a**) Specimen 3; (**b**) Specimen 4.

**Figure 20:**
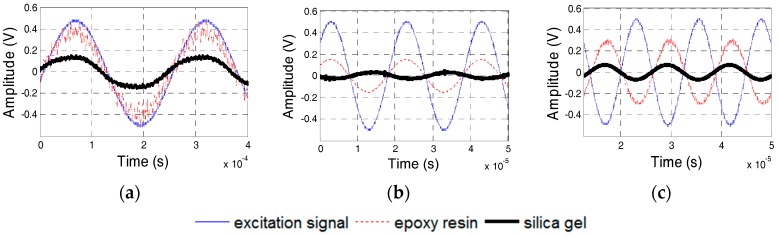
PZT transducer signal of Specimen 3. (**a**) 4 kHz Excitation Frequency; (**b**) 30 kHz Excitation Frequency; and, (**c**) 55 kHz Excitation Frequency.

**Figure 21:**
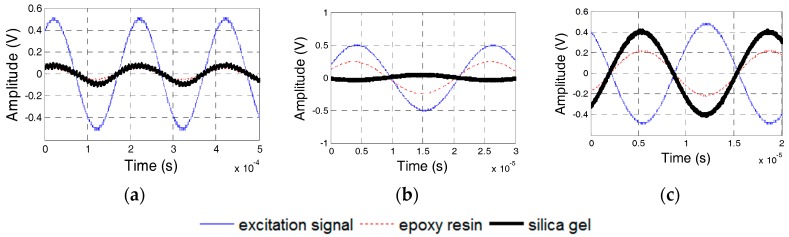
PZT transducer signals of Specimens 4. (**a**) 4 kHz Excitation Frequency; (**b**) 30 kHz Excitation Frequency; and, (**c**) 55 kHz Excitation Frequency.

Epoxy resin and silicon are used as the waterproof layer in transducers. For Specimens 3 and 4, the dynamic properties of the transducers with a waterproof layer of epoxy resin and silicon were tested. The phase difference of the two transducers in Specimens 3 and 4 is shown in Figure 19, which reveals the strong similarity between the experimental results and the numerical results. The natural frequencies of the PZT transducers in Specimens 3 and 4 should be 16 and 48 kHz, respectively. Figure 20 and Figure 21 show the excitation signal and PZT transducer signals of Specimens 3 and 4, respectively. When the excitation frequency is 30 kHz, then the signal of the transducer with a silicon waterproof layer changes by *π*, which illustrates that the natural frequency of the transducer is less than 30 kHz. Hence, the natural frequency of the transducer with a silicon waterproof layer is 16 kHz, whereas the natural frequency of transducer with an epoxy resin waterproof layer is 48 kHz. 

### 5.4. Effect of the Thickness of the Waterproof Layer 

**Figure 22:**
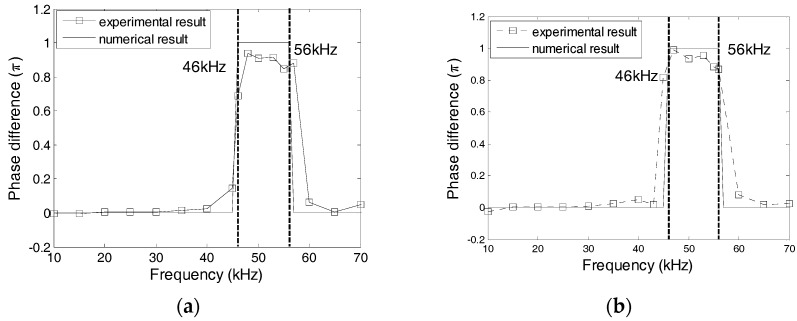
Phase difference between the PZT sensors in Specimens 5 and 6. (**a**) Specimen 5; (**b**) Specimen 6.

**Figure 23:**
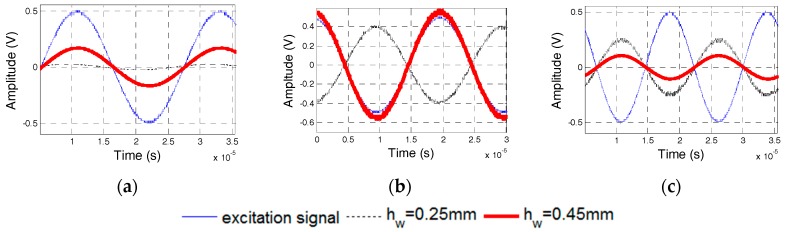
PZT transducer signals of Specimen 5. (**a**) 20 kHz Excitation Frequency; (**b**) 50 kHz Excitation Frequency; and, (**c**) 65 kHz Excitation Frequency.

**Figure 24:**
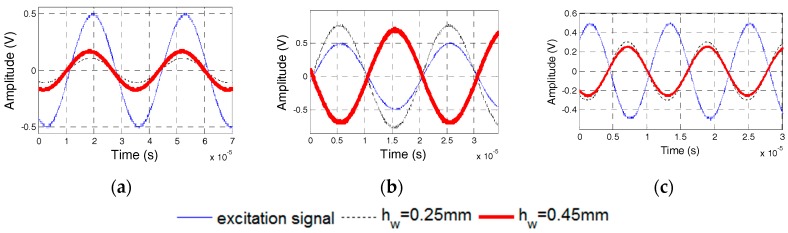
PZT transducer signals of Specimen 6. (**a**) 20 kHz Excitation Frequency; (**b**) 50 kHz Excitation Frequency; and (**c**) 65 kHz Excitation Frequency.

Specimens 5 and 6, whose waterproof layers are 0.25-mm and 0.45-mm thick, respectively, were manufactured to test the dynamic properties of PZT sensors with different thicknesses of the waterproof layers. Figure 22 shows the phase difference of two PZT transducers in Specimens 5 and 6. The natural frequencies of the PZT sensors in Specimens 5 and 6 were 46 and 56 kHz, respectively. Figure 23 and Figure 24 show the excitation signal and PZT transducer signal when the excitation frequency was 20, 50, and 65 kHz. With an increase in the excitation frequency, the signal phase of the transducer with the 0.45-mm-thick waterproof layer changes first, which illustrates that the natural frequency for the transducer with the 0.45-mm-thick waterproof layer is 46 kHz, and the other one is 56 kHz. The experimental results are similar to the numerical results. The natural frequency of the transducer increases with the increasing thickness of the waterproof layer.

### 5.5. Effect of the PZT Thickness 

**Figure 25:**
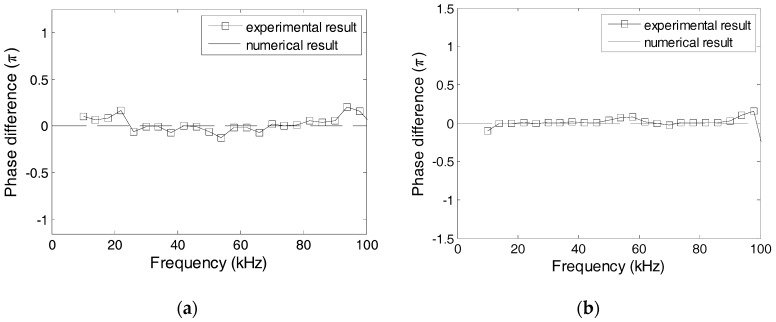
Phase difference between PZT-1 and PZT-2 for Specimens 7 and 8. (**a**) Specimen 7; (**b**) Specimen 8.

Specimens 7 and 8, which were made by the PZT transducer with thicknesses of 0.5 and 1 mm, illustrate the dynamic properties that are caused by the PZT transducer thickness. Figure 25 shows the phase difference between the two PZT sensors in Specimens 7 and 8. The numerical results are similar to the experimental results. The PZT thickness does not affect the natural frequency of the transducer considerably.

## 6. Conclusions

This paper proposes a dynamic mechanic model of the embeddable PZT transducers with waterproof layers and protecting layers. The proposed model is validated by the FEM. Then, the effects of the protecting layer, waterproof layer, and PZT patch on the dynamic property are analyzed. Finally, this analysis was verified based on experiments of the dynamic stress transfer of PZT transducers. Based on the proposed model, the factors that are influencing the dynamic property of the embeddable PZT transducers, which include the material and thickness of the protecting layer, the material and thickness of the waterproof layer, and the thickness of the PZT, are analyzed. The results show that the excitation frequency can significantly affect the stress transfer of the PZT transducer in terms of both amplitude and signal phase. The natural frequency in the poling direction for the PZT transducer is affected by the material properties and the thickness of the waterproof and protecting layers. 

Based on the dynamic transfer model that is proposed in this paper, by choosing the specific thickness and materials of the PZT patch, the waterproof layer and protecting layer, the embeddable PZT transducers can be more sensitive, or has a better stability of dynamic stress transfer property in a special frequency range of stress responses. Therefore, the findings in this paper provide a scientific basis to design embeddable PZT transducers with special functions.

## Figures and Tables

**Table 1:** Parameters of the PZT: waterproof layer and protecting layer.

PZT Patch		Waterproof Layer		Protecting Layer	
*h_p_*	0.001 m	*h_w_*	0.0001 m	*h_c_*	0.015 m
*ρ_p_*	7600 kg/m^3^	*ρ_w_*	1105 kg/m^3^	*ρ_c_*	2700 kg/m^3^
*E_p_*	7.65 × 10^10^ N/m^2^	*E_w_*	3 × 10^9^ N/m^2^	*E_c_*	5 × 10^10^ N/m^2^

**Table 2:** Parameters of different protecting layer materials.

Material	Destiny (kg/m^2^)	Elastic Modulus (GPa)	*E_c_/ρ_c_*
concrete	2500	23	9.20 × 10^6^
brass	8000	70	11.41 × 10^6^
granite	3000	43	14.54 × 10^6^
marble	2700	55	20.37 × 10^6^
Carbon steel	7400	206	27.83 × 10^6^

**Table 3:** Test specimens.

Specimen	Transducers	Protecting Layer Material	Waterproof Layer Material	Waterproof Layer Thickness	PZT Patch Thickness
**Specimen 1**	SA-1	carbon steel	epoxy resin	0.4 mm	1 mm
	SA-2	marble	epoxy resin	0.4 mm	1 mm
**Specimen 2**	SA-1	marble	epoxy resin	0.4 mm	1 mm
	SA-2	plexiglas	epoxy resin	0.4 mm	1 mm
**Specimen 3**	SA-1	marble	Silicon	0.4 mm	1 mm
	SA-2	marble	epoxy resin	0.4 mm	1 mm
**Specimen 4**	SA-1	marble	Silicon	0.4 mm	1 mm
	SA-2	marble	epoxy resin	0.4 mm	1 mm
**Specimen 5**	SA-1	marble	epoxy resin	0.25 mm	1 mm
	SA-2	marble	epoxy resin	0.45 mm	1 mm
**Specimen 6**	SA-1	marble	epoxy resin	0.25 mm	1 mm
	SA-2	marble	epoxy resin	0.45 mm	1 mm
**Specimen 7**	SA-1	marble	epoxy resin	0.45 mm	0.5 mm
	SA-2	marble	epoxy resin	0.45 mm	1 mm
**Specimen 8**	SA-1	marble	epoxy resin	0.45 mm	0.5 mm
	SA-2	marble	epoxy resin	0.45 mm	1 mm
